# Comprehensive data mining reveals RTK/RAS signaling pathway as a promoter of prostate cancer lineage plasticity through transcription factors and CNV

**DOI:** 10.1038/s41598-024-62256-z

**Published:** 2024-05-22

**Authors:** Guanyun Wei, Xu Zhang, Siyuan Liu, Wanxin Hou, Zao Dai

**Affiliations:** 1 Research Center for Intelligent Information Technology, Nantong University, Nantong, China; 2 Co-Innovation Center of Neuroregeneration, School of Life Sciences, Nantong Laboratory of Development and Diseases, Nantong University, Nantong, China; 3 Clinical Medical Research Center, Jiangnan University Medical Center, Wuxi No.2 People’s Hospital, Affiliated Wuxi Clinical College of Nantong University, Wuxi, China; 4 School of Life Sciences, Nantong University, Nantong, China

**Keywords:** Cancer, Prostate cancer, Data integration, Data mining

## Abstract

Prostate cancer lineage plasticity is a key driver in the transition to neuroendocrine prostate cancer (NEPC), and the RTK/RAS signaling pathway is a well-established cancer pathway. Nevertheless, the comprehensive link between the RTK/RAS signaling pathway and lineage plasticity has received limited investigation. In particular, the intricate regulatory network governing the interplay between RTK/RAS and lineage plasticity remains largely unexplored. The multi-omics data were clustered with the coefficient of argument and neighbor joining algorithm. Subsequently, the clustered results were analyzed utilizing the GSEA, gene sets related to stemness, multi-lineage state datasets, and canonical cancer pathway gene sets. Finally, a comprehensive exploration of the data based on the ssGSEA, WGCNA, GSEA, VIPER, prostate cancer scRNA-seq data, and the GPSAdb database was conducted. Among the six modules in the clustering results, there are 300 overlapping genes, including 3 previously unreported prostate cancer genes that were validated to be upregulated in prostate cancer through RT-qPCR. Function Module 6 shows a positive correlation with prostate cancer cell stemness, multi-lineage states, and the RTK/RAS signaling pathway. Additionally, the 19 leading-edge genes of the RTK/RAS signaling pathway promote prostate cancer lineage plasticity through a complex network of transcriptional regulation and copy number variations. In the transcriptional regulation network, TP63 and FOXO1 act as suppressors of prostate cancer lineage plasticity, whereas RORC exerts a promoting effect. This study provides a comprehensive perspective on the role of the RTK/RAS pathway in prostate cancer lineage plasticity and offers new clues for the treatment of NEPC.

## Introduction

Prostate cancer is a prevalent malignancy that predominantly affects elderly males^1^. In 2016, 95% of prostate cancer patients were over 60 years old, and 51.2% of those were over 80 years old. The global incidence of prostate cancer in 2018 reached 1.3 million new cases, and there were 355,000 deaths attributed to the disease. This positions prostate cancer as the second most common cancer worldwide and the fifth leading cause of cancer-related deaths among men^2^.

Lineage plasticity has emerged as a critical mechanism contributing to treatment resistance in prostate cancer. Androgen deprivation therapy is a standard treatment due to the dependency of prostate cancer cells on the androgen receptor (AR)^3,4^. In clinical practice, some castration-resistant prostate cancer patients have tumor cells that exhibit both morphological features of neuroendocrine cancer and mixed luminal and basal cell characteristics upon recurrence^5^. This ability to transition from epithelial cells to neuroendocrine cells is lineage plasticity^6^. The acquisition of prostate cancer cell lineage plasticity can lead to cells transforming into stem-like and multilineage states, differentiating into new or distinct lineages, undergoing reprogramming to transform into a novel type of cell that can survive without the need for AR, and leading to the acquisition of AR-independent characteristics through lineage transformation^7^. There has been recent progress in the research on prostate cancer lineage plasticity. Reports indicate that the loss of tumor suppressor functions like TP53 and RB1 promotes the transition of AR-dependent luminal epithelial cells to AR-independent basal-like cells, and EZH2 inhibitors have been found to reverse this lineage transition and to restore sensitivity to androgen deprivation therapy^8^. Additionally, SOX2, through the JAK-STAT signaling pathway, can facilitate the conversion of AR-dependent prostate cancer cells into a multi-lineage state^7,9^. FOXA2 can induce the transition of multi-lineage state cells to Neuroendocrine Prostate Cancer (NEPC) through the KIT pathway^10^. ASCL1, activated downstream of the ROR2/CREB signaling pathway, contributes to prostate cancer lineage plasticity^11^. Specific SWI/SNF complexes are associated with PCa progression and may play a role in treatment resistance^12^. Moreover, epigenetic factors such as chromatin modifications and DNA methylation also play a role in promoting lineage plasticity^13^. Indeed, lineage plasticity is not confined to prostate cancer, as this phenomenon has been noted in both lung and breast cancer^14,15^. However, the research on lineage plasticity remains incomplete, there are limited clinical trials addressing these challenges, and the prognosis for patients continues to be bleak.

Public databases offer rich sources of various omics data related to prostate cancer. This study leverages these to analyze and explore lineage plasticity of prostate cancer. We conducted an analysis of multi-omics data from TCGA and SRA databases, including AR ChIP-seq, transcriptomic, single-cell transcriptomic, lncRNA, SNV, CNV, DNA methylation, and clinical data of PCa patients to identify key gene sets related to prostate cancer. Then, annotation and clustering analysis were applied to these gene sets, resulting in 6 function modules. Based on the TCGA, NEPC, CRPC cohort data, and data on prostate cancer cell stemness and lineage plasticity, it was discovered that function module 6 is more closely associated with CRPC and is related to cancer stemness and lineage plasticity. Analysis of Module 6 in relation to 10 oncogenic signaling pathways (cell cycle, Hippo signaling, Myc signaling, Notch signaling, oxidative stress response/Nrf2, PI-3-Kinase signaling, receptor-tyrosine kinase (RTK)/RAS/MAP-Kinase signaling, TGFb signaling, p53, and b-catenin/Wnt signaling)^16^, revealed that it is positively correlated with 3 of these cancer pathways (cell-cycle, p53, and RTK/RAS). The RTK/RAS pathway plays a crucial role in the progression, extraprostatic extension, and development of castration-resistant prostate cancer (CRPC), and contributes to prostate cancer lineage plasticity^11,17^.

Measuring the extent of the RTK/RAS pathway's contribution to lineage plasticity in prostate cancer is challenging. Through a series of computational analyses, we explored the connections between the marker gene set of multi-lineage state cells, prostate cancer samples, and the gene set associated with the RTK/RAS signaling pathway. The findings revealed that the RTK/RAS signaling pathway promotes lineage plasticity. Transcription factors (TFs) and genomic variations are molecular driving factors of prostate cancer lineage plasticity^18^. This study unveiled that both copy number variations (CNVs) and TFs contribute to the RTK/RAS pathway in prostate cancer lineage plasticity. Particularly, TFs TP63 and FOXO1 suppress and RORC drives prostate cancer lineage plasticity through the RTK/RAS pathway.

The investigation into prostate cancer lineage plasticity and its driving factors is important for combating cancer resistance. This work revealed that CNVs within the RTK/RAS pathway, along with their upstream TFs, drive lineage plasticity of prostate cancer. We also identified a set of potential target gene options for prostate cancer patients who have developed treatment resistance. Moreover, this study offers novel insights and references for drug resistance research for other forms of cancer.

## Results

### Differential expression genes, prognostic lncRNAs, and extraprostatic extension related genes were obtained through prostate *cancer* expression data analysis

**Figure 1:**
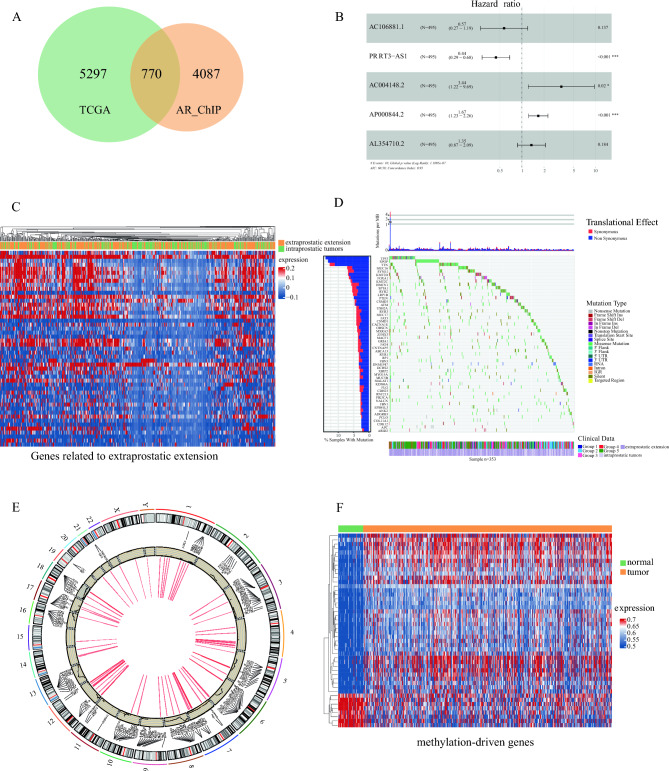
Results of prostate cancer transcriptome and genomic data analyses. (**A**) Venn diagram of androgen receptor (AR) target genes and differentially expressed genes in prostate cancer. (**B**) Forest plot of 5 lncRNAs in prostate cancer. (**C**) Heatmap of differentially expressed genes between extraprostatic extension and intraprostatic tumors. (**D**) Prostate cancer mutation waterfall plot. (**E**) Prostate cancer CNVs and loops circos plot. (**F**) Prostate cancer methylation-driven gene heatmap.

There are 770 DEGs found to be regulated by AR in prostate cancer through intersecting AR Chip-seq data from the SRA and TCGA databases (Fig. 1A). These DEGs play a significant role in the progression of prostate cancer, for example: *TIMP3*^19^, *HSD17B3*^20^, *SRD5A2*^21^, *FN1*^22^, *CYP19A1*^23^, *SGK1*^24^, SCNN1A^25^, RND3^26^, FASN^27^, CDK15^28^, SOX5^29^ (additional details can be found in the Supplementary Table S1). GO and KEGG enrichment analyses were performed on these genes (Supplementary Tables S2 and S3) and all annotated genes are provided in Supplementary Table S4.

Then, 5 lncRNAs that were associated with the prognosis of prostate cancer was identified by univariate and multivariate Cox analyses (Supplementary Fig. S1) based on lncRNA transcriptome data from the TCGA prostate cancer database (Fig. 1B), and the genes interacting with these lncRNAs were annotated (Supplementary Tables S5–S7). The predictive model was defined as the linear combination of the expression levels of the 5 lncRNAs, which were weighted as follows: survival risk score = ((− 0.56041) × AC106881.1 + (0.81588) × PRRT3-AS1 + (1.234135) × AC004148.2 + (0.511379) × AP000844.2 + (0.297335) × AL354710.2) (*p* = 1.11e−07; C-index = 0.95). The threshold of risk score was 0.8789252 with linear model fits. PRRT3-AS1 has the capability to effectively inhibit the proliferation of prostate cancer cells, induce cell apoptosis and autophagy^30^, and it is a biomarker and therapeutic target for skin cutaneous melanoma^31^. AP000844.2 is involved in the PCa’s early prognosis and risk stratification^32^, and its expression exhibits significant differences among various aging-related subtypes in prostate cancer patients^33^. Additionally, Huang et al. (2022) also identified the significance of AP000844.2 in their analysis of the role of lncRNAs in prostate cancer^34^. AC106881.1 is an antisense RNA of UNC5C, which is involved in neuronal development^35^. AC004148.2 is one of the lncRNAs in the immune-related lncRNA risk model for the prognosis of head and neck squamous cell carcinoma patients^36^. From the analysis of differential expression between extraprostatic extension and intraprostatic tumors, a total of 372 genes associated with Gleason grading were identified (Fig. 1C). More detailed gene information and annotation results can be found in Supplementary Tables S8–S10.

### Acquisition and annotation of SNV, CNV, and methylation-driven genes in prostate *cancer*

The analysis of prostate cancer SNV data indicates that (Fig. 1D), in high mutation samples (TP53 + SPOP + TTN), samples with TP53 mutations are significantly enriched in those with Gleason grade 5 (8.28e-03) and in samples with extraprostatic extension of prostate cancer (1.756173e-34). The corresponding DEGs associated with these mutations can be found in Supplementary Table S11, and the results of GO and KEGG analyses are presented in Supplementary Tables S12 and S13.

Furthermore, the visualization of prostate cancer CNVs and loops was performed, depicting their locations on the chromosomes (Fig. 1E). BCL2^37^, ETV6^38^, and CDKN1B^39^ are important genes in the occurrence and development of prostate tumors, experiencing not only copy number deletions but also appearing in loops. The detailed list of DEGs between CNV and non-CNV samples can be found in Supplementary Table S14, and the annotation analysis information for DEGs can be accessed in Supplementary Tables S4 and S15–S16.

Methylation-driven genes were also identified (Fig. 1F) and a detailed list of these can be found in Supplementary Table S17 and the corresponding annotation information can be accessed in Supplementary Tables S4 and S18–19.

Typically, a biological process is accomplished by a group of genes working together, while a biological phenotype is regulated by multiple biological processes. Therefore, mining the results of GO and KEGG enrichment analysis based on multi-omics data enabled the assembly of prostate cancer-related functions or pathways into a larger biological context, leading to a better understanding of the biological questions related to prostate cancer phenotypes. CRPC (Castration-Resistant Prostate Cancer) and NEPC (Neuroendocrine prostate cancer) are two classic phenotypes of prostate cancer. By integrating three cohort datasets from TCGA, CRPC, and NEPC, and quantifying the CRPC and NEPC scores in TCGA, it was found that samples in TCGA with T3b, T4, N1 stages, Gleason grade group 5, and those that received drug treatments also exhibited significantly higher CRPC and NEPC scores (Supplementary Fig. S2).

### Six functional modules were identified through a comprehensive analysis of DEGs, prognostic lncRNAs, extraprostatic extension related genes, SNV, CNV, and methylation-driven genes in prostate cancer

**Figure 2:**
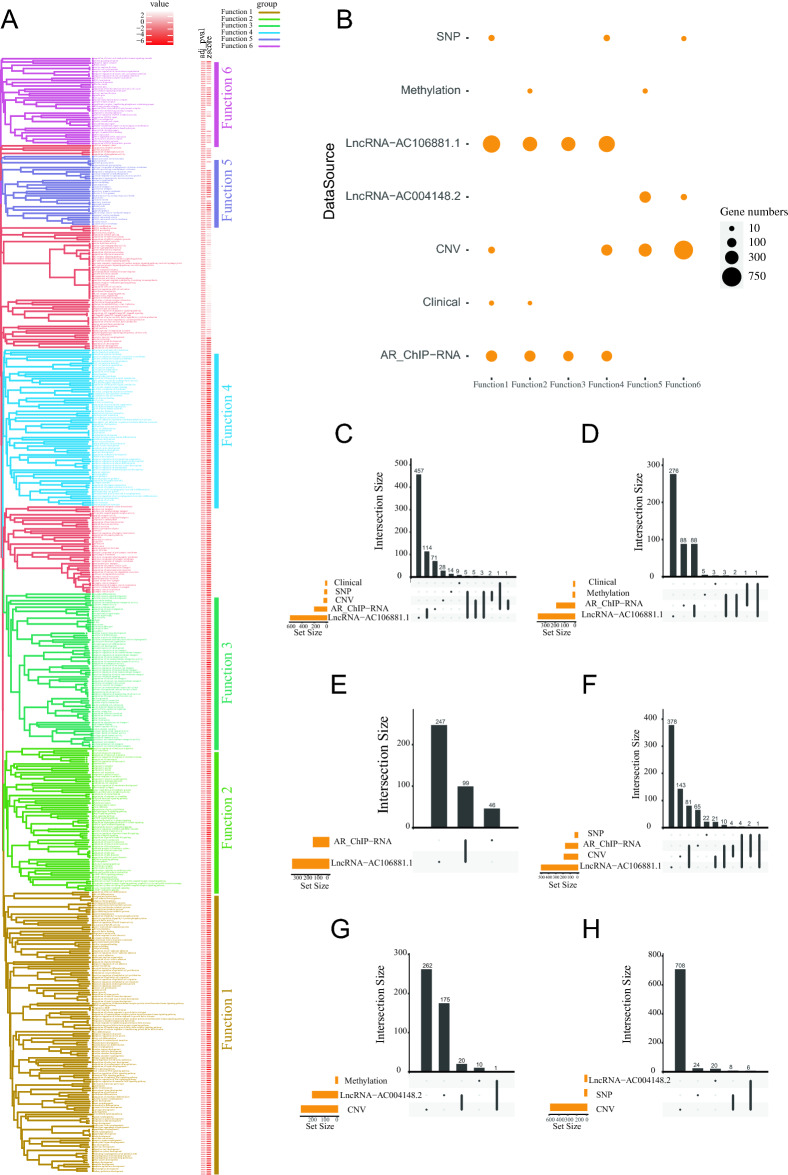
Cluster dendrogram based on GO and KEGG enrichment analysis results. (**A**) Neighbor joining cluster tree of GO and KEGG enrichment analysis results for prostate cancer multi-omics data. (**B**) The relationship between Function Modules 1–6 and the six datasets is summarized. (**C–H**) Statistics of prostate cancer multi-omics data corresponding to module function1—module function6.

Further clustering analysis was performed on the annotation results of the six datasets on prostate cancer, including the DEGs, genes regulated by prognostic lncRNAs, genes associated with extraprostatic extension, DEGs between mutation and non-mutation prostate cancer samples, DEGs between CNV and non-CNV prostate cancer samples, and methylation-driven genes. These pathways and functions were classified into six Functional Modules (Fig. 2A). After scoring TCGA, CRPC, and NEPC using six functional modules, it was discovered that functional modules 5 and 6 differ from modules 1–4 and are closely associated with the CRPC phenotype. Moreover, CRPC and NEPC characteristics exhibit a positive correlation with functional module 6 (Supplementary Fig. S3). Function Modules 1–4 encompass processes related to cancer occurrence and development, including the Wnt signaling pathway, positive regulation of epithelial cell proliferation, positive regulation of endothelial cell proliferation, endothelial cell migration, Ras signaling pathway, muscle cell development, cell adhesion molecules, and cell–cell junction. Function Module 5 includes biological processes related to the microenvironment and epithelial-mesenchymal transition (EMT), such as responses to endoplasmic reticulum stress and protein glycosylation hydrolase activity, acting on glycosyl bonds. Notably, Function Module 6 includes entries related to stemness, such as the regulation of G2/M transition of mitotic cell cycle, mitotic nuclear division, and response to ionizing radiation. It is important to highlight that Function Module 6 has a low Z-score (lighter color bar), indicating a significant upregulation of the pathway.

In addition, a protein–protein interaction network was constructed based on 300 genes obtained from the intersection of the six datasets (Supplementary Fig. S4A). A core network consisting of 19 genes was identified, and most genes in the core network have been reported to play important roles in prostate cancer, especially *TPM1, ITGB3*, and *CALD1* (Supplementary Fig. S4B). RT-qPCR validation was performed on these three genes, which revealed their high expression in prostate cancer tissues (Supplementary Fig. S4C).

The relationship between Function Modules 1–6 and the six datasets is summarized in Fig. ure 2B. All six gene-sets are involved in Function Modules 1–4, while the gene-sets of CNV, lncRNA and Methylation are involved in Function Module 5. The gene-sets of lncRNA, SNV, and CNV are involved in Function Module 6. More detailed information about the six functional modules and their source data is presented in Fig. 2C–H. Some genes in the Functional Modules are derived from multiple datasets. A total of 42 genes in the six Functional Modules were investigated, and 26 of these genes exhibit expression in prostate cancer cells or tissues (Supplementary Fig. S5). Among these genes, *SOX5*^29^*, PRKCB*^40^*, VCL*^41^*, ARSB*^42^*, TF (Transferrin)*^43,44^*, APC*^45^*, PTEN*^46^*, TP53*^47^*, BRCA1*^48^*, DCP2*^49^*, DCUN1D2*^50^*, MKLN1*^51^*, LPAR1*^52^, *EFNA5 and SORBS1*^53,54^ have been reported play important roles in prostate cancer. *COL14A1*^55^*, ANGPT1*^56^*, SERPINF1*^57^*,* GJA1^58^, SMC2^59^, PCID2^60^, FBXL8^61^, and FBXL17^62^ have also been reported in other cancers. KCNS2, MYOCD^63^, and DCAF8^64^ are involved in other biological processes. These results suggest that the genes identified in our study but not previously reported to be involved in prostate cancer may be candidate genes in prostate cancer.

### GSEA analysis of the Function Modules suggested that the stemness of prostate *cancer* cells primarily originates from genetic mutations

**Figure 3:**
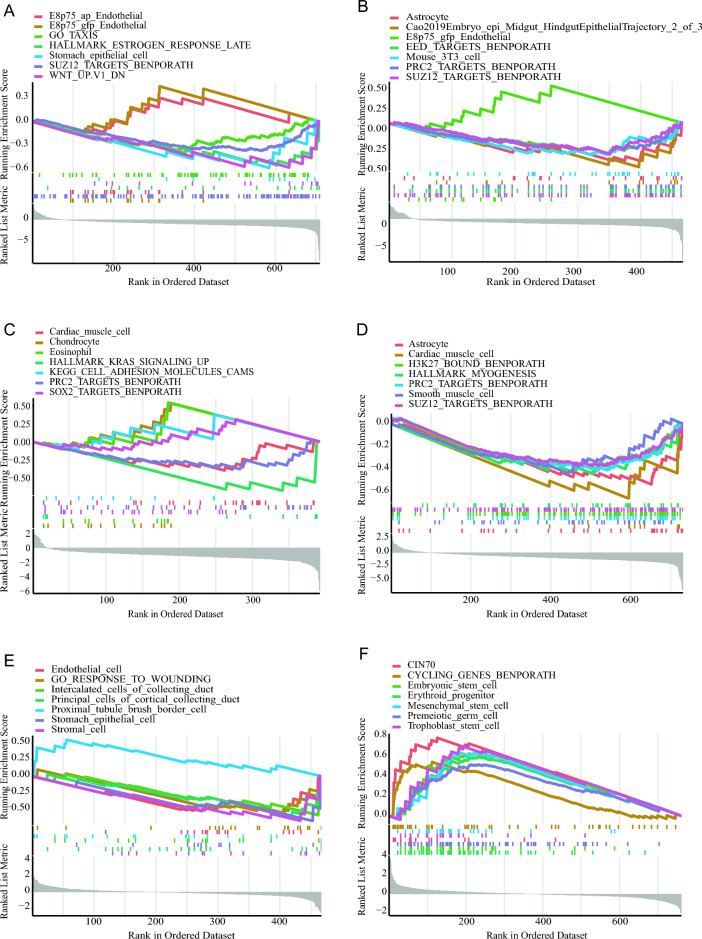
The analysis results of Gene Set Enrichment Analysis (GSEA) for each cluster in the clustering tree. (**A**–**F**) correspond to the GSEA analysis results for module function1—module function6, respectively.

GSEA analysis was performed on the six functional modules (Fig. 3). The results revealed that Function Module 1–5 (Fig. 3A–E) were negatively enriched with tumor stem cell characteristics (SUZ12_TARGETS_BENPORATH or PRC2_TARGETS_BENPORATH). In addition, Function Module 5 (Fig. 3E) also showed negative enrichment with epithelial, endothelial cells, and wound response functions, suggesting it represents a complex tumor microenvironment that may be associated with EMT in prostate cancer. Surprisingly, in contrast to Function Modules 1–5, Function Module 6 exhibited a significant positive correlation with stem cells, meaning that there is a close relationship between it and cell stemness (Fig. 3F). Moreover, it is noteworthy that the primary dataset for Function Module 6 is derived from CNV analysis. This result provides compelling evidence that CNV is helpful for the stemness of prostate cancer cells. This aligns with previous research that also found that high CNV contributes to the stemness of prostate cancer cells^65^.

### Function Module 6 is positively correlated with lineage plasticity in prostate cancer as shown through integrative analysis of multiple datasets, which provides a valuable means for assessing the lineage plasticity in samples

**Figure 4:**
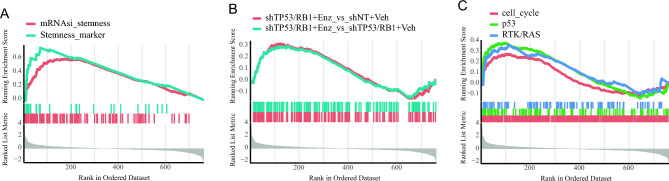
The RTK/RAS pathway promotes prostate cancer lineage plasticity. (**A**) Prostate cancer stemness is positively enriched in module function6 cluster. (**B**) Transcriptional data of prostate cancer multi-lineage states are positively enriched in module function6. (**C**) The cell_type, p53, and RTK/RAS pathway are positively enriched in function6/pathway6 cluster.

Multi-lineage states represent important stages of cancer cell differentiation characterized by both stemness and multi-lineage potential. Evaluating lineage plasticity in prostate cancer is currently a research hotspot and major challenge. Based on GSEA analysis and datasets associated with stem cell-related genes^66,67^, our study revealed a positive correlation between the Function Module 6 and stemness (Fig. 4A). Leveraging GSEA analysis and multi-lineage states datasets^7^, our investigation unveiled a positive relationship between the Function Module 6 and multi-lineage states (Fig. 4B). Moreover, employing GSEA analysis along with datasets related to classic cancer driver pathways^16^, our findings demonstrated that the Function Module 6 is positively associated with three cancer pathways (cell-cycle, p53, and RTK/RAS) (Fig. 4C), thus, a substantial connection between Function 6 and these biological aspects was confirmed. This finding suggests that the Function Module 6 can serve as a functional module for assessing lineage plasticity in prostate cancer.

### CNVs and TFs contribute to the involvement of RTK/RAS pathway genes in prostate *cancer* lineage plasticity

**Figure 5:**
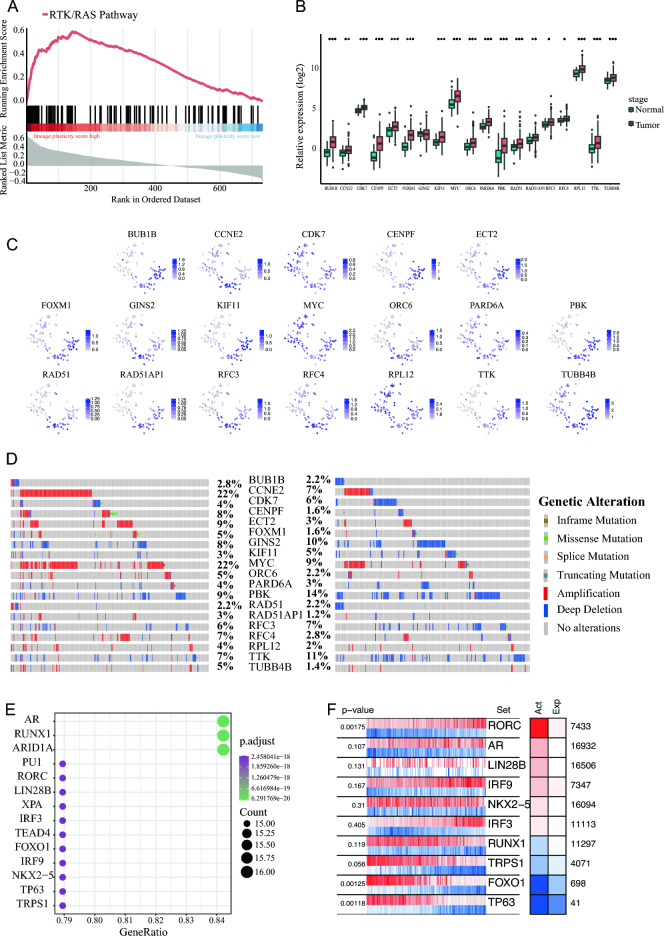
Analysis results of the expression, CNVs, and transcription factor activity of the 19 leading edge genes in RTK/RAS signaling pathway. (**A**) The RTK/RAS signaling pathway is positively enriched in high lineage plasticity score samples of prostate cancer. (**B**) Upregulation of the 19 leading edge genes in prostate cancer RNA-seq data from the TCGA database. (**C**) The expression of 19 leading-edge genes in prostate cancer single-cell RNA-seq data, with the x-axis and y-axis representing t-SNE1 and t-SNE2, respectively. (**D**) Copy number variations of the 19 leading edge genes in the TCGA prostate cancer cohort (right) and the SU2C prostate cancer cohort (left). (**E**) The transcription factors that top enrichment leading edge genes in the GPSAdb database. (**F**) Transcription factors have transcription active in prostate cancer.

Recent research has highlighted the involvement of RTK in prostate cancer lineage plasticity^11^, and our analyses have indicated Function Module 6 is positively correlated with prostate cancer lineage plasticity and RTK/RAS (Fig. 4). Thus, our research centered on RTK/RAS and aims to systematically delve into the intricacies of the pathway in relation to prostate cancer lineage plasticity through bioinformatics analysis. In our approach, we overcame the challenge of quantifying the relationship between pathways and lineage plasticity by employing ssGSEA and WGCNA method calculations to derive correlation values between all genes within the Function Module 6 and the lineage plasticity of prostate cancer. We further subjected all genes in the RTK/RAS signaling pathway to GSEA analysis for their lineage plasticity, revealing that it indeed enhances lineage plasticity (Fig. 5A).

Then, we analyzed the 19 LEGs (*BUB1B, CCNE2, CDK7, CENPF, ECT2, FOXM1, GINS2, KIF11, MYC, ORC6, PARD6A, PBK, RAD51, RAD51AP1, RFC3, RFC4, RPL12, TTK*, and *TUBB4B*) which drive the prostate cancer lineage plasticity through the RTK/RAS signaling pathway as identified using GSEA. These LEGs exhibited significant upregulation in prostate cancer samples from the TCGA database (Fig. 5B and Supplementary Table S20), and their expression was also detected in single-cell transcriptome sequencing of the prostate cancer cell line LNCaP^68^ (Fig. 5C). Moreover, these 19 genes displayed varying levels of CNVs in both the TCGA and SU2C prostate cancer cohorts (Fig. 5D). Previous research indicates that some of the LEGs, including *BUB1B*^69^*, CDK7*^70^*, CENPF*^71^*, FOXM1*^72^*, KIF11*^73,74^*, ORC6*^75^*, PBK*^76^*, RAD51*^77^*, TTK*^78^*, CCNE2*^79^*,* and *MYC*^80^, have previously been reported to be associated with prostate cancer, particularly in relation to metastasis. Additionally, ECT2^81^, GINS2^82^, PARD6A^83^, RAD51AP1^84^, RFC3^85^, RFC4^86^, and TUBB4B^87^ have been reported to function in other cancer occurrences. All these LEGs contribute to lineage plasticity.

Based on the transcriptome data of different TF knockdowns from the GPSAdb database^88^, a total of 194 TFs were enriched (Supplementary Table S21) and hence impact the expression of LEGs. The TFs that for the top enrichment LEGs were identified (Fig. 5E), and among these the 10 with transcription active in prostate cancer were selected (Fig. 5F). These indicate a closer correlation between the 10 TFs and the lineage plasticity of prostate cancer. Moreover, all 10 TFs, including RORC^89^, AR^90^, LIN28B^91^, IRF9^92^, NKX2-5^93^, IRF3^94,95^, RUNX1^96^, TRPS1^97^, FOXO1^98^, and TP63^8^, have been reported to play roles in prostate cancer, especially in castration-resistant prostate cancer. This finding further confirms the reliability of our study. It is worth noting that TP63, FOXO1, and RORC exhibit significant transcription activity (*p*-value < 0.05), with the transcription activity of TP63 and FOXO1 being reduced and RORC increased (Fig. 5F). It is established that TP63 regulates the expression of p53, which can inhibit prostate cancer lineage plasticity, suggesting that TP63 could potentially contribute to the inhibition of prostate cancer lineage plasticity^8^. FOXO1 has been shown to restrain the migration and invasion of prostate cancer cells^98^. On the other hand, RORC drives the expression of AR in prostate cancer^89^. Based on all the information presented above, TP63 and FOXO1 suppress prostate cancer lineage plasticity through the RTK/RAS pathway, whereas RORC drives prostate cancer lineage plasticity through RTK/RAS pathway.

In summary, the research indicates that both CNVs and TFs contribute to the involvement of the RTK/RAS pathway in prostate cancer lineage plasticity.

## Discussion

**Figure 6:**
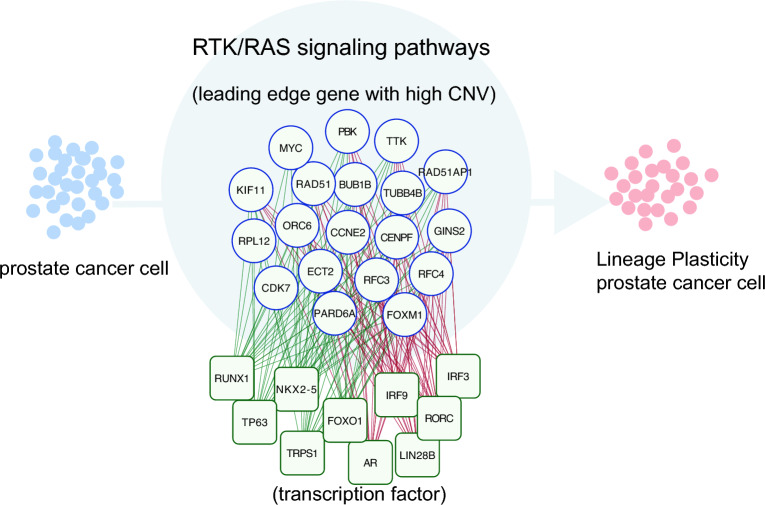
Schematic diagram of the RTK/RAS pathway promotes prostate cancer lineage plasticity.

Treatment for advanced prostate cancer patients remains undiscovered. After acquiring lineage plasticity, prostate cancer cells progress into neuroendocrine prostate cancer. Moreover, lineage plasticity also occurs in other malignant tumors. The study of prostate cancer lineage plasticity and its driving factors is a focus point in prostate cancer research, which has significant importance in advancing our understanding of cancer drug resistance and related research. Our analyses have revealed that the RTK/RAS signaling pathway promotes prostate cancer lineage plasticity. A total of 19 LEGs with high numbers of CNVs significantly contribute to this process, and the TFs that influence the expression of these genes were identified. TFs regulating the same gene exhibited contrasting effects: some suppressed prostate cancer lineage plasticity and others promoted it (Fig. 6). This provides a range of potential targets for treatment and novel insights for investigating prostate cancer lineage plasticity.

Here, we aimed to explore the critical factors in the development of prostate cancer from a more comprehensive and systematic perspective. The key gene sets associated with prostate cancer across various omics profiles were obtained from the TCGA and SRA databases, including data from AR ChIP-seq, transcriptomic, single-cell transcriptomic, lncRNA, SNV, CNV, DNA methylation, and clinical data for prostate cancer patients (Fig. 1). Network analysis was performed on the intersection of 300 genes from these six datasets, and qRT-PCR validation confirmed the 3 top hub genes (TPM1, ITGB3, and CALD1) in the network are expression in prostate cancer tissues (Supplementary Fig. S4). The 3 top hub genes represent significant alterations at every level in prostate cancer and have been reported to play important roles in the cancer^65,99,100^. Then, six function modules were obtained through a neighbor joining algorithm based on the GO and KEGG enrichment analysis of prostate cancer multi-omics data (Fig. 2A). Cellular proteomics analysis was performed on the intersecting genes of the 6 function modules, and the results indicated that most of these exhibited protein expression in prostate cancer (Fig. S5). Notably, APC, PTEN, TP53, and BRCA1, all tumor suppressor genes, were intersecting genes in the SNV and CNV datasets, and these are also in Function Module 6. APC acts as an antagonist of the Wnt signaling pathway, and there is a strong correlation between APC methylation and prostate cancer^45^. Loss of PTEN function is one of the most common driver events in the development of prostate cancer^46^. TP53 mutations are closely associated with tumor progression in prostate cancer, and mutations are widespread in various cancer types^47^. Pathogenic variants of BRCA1 have been extensively studied for their association with the risk of prostate cancer^48^. This suggests Function Module 6 and CNVs play crucial roles in the development of prostate cancer. Our previous work has also indicated the involvement of CNV in prostate cancer development^67^.

Further analysis of the six functional modules revealed that the Function Module 6 is the only one with significant positive correlation with stem cell characteristics, while the Function Module 1–5 are negatively enriched with tumor stem cell (Fig. 3). Moreover, Function Module 6 is also positively correlated with the prostate cancer multi-lineage state (Fig. 4AB), suggesting a positive correlation between it and prostate cancer lineage plasticity as the coexistence of multi-lineage states and stemness is a characteristic of a lineage plasticity cell. Notably, our analysis indicates a positive correlation between Function Module 6 and the RTK/RAS signaling pathway (Fig. 4C), which is one of the classical cancer pathways and has recently been reported to play a role in prostate cancer lineage plasticity^11^.

By integrating multiple computational methods, it has been confirmed that the RTK/RAS signaling pathway is significantly enriched in samples with high scores for prostate cancer lineage plasticity (Fig. 5A). All LEGs driving the RTK/RAS signaling pathway to promote prostate cancer lineage plasticity exhibit significantly higher expression in prostate cancer samples compared to normal samples (Fig. 5B), and they display high expression in scRNA-seq data for prostate cancer (Fig. 5C). Most of the LEGs, including BUB1B, CDK7, CENPF, FOXM1, KIF11, ORC6, PBK, RAD51, TTK, CCNE2, and MYC, have previously been reported to be associated with prostate cancer, particularly in the context of metastasis, and other LEGs, including ECT2, GINS2, PARD6A, RAD51AP1, RFC3, RFC4, and TUBB4B, have also been reported to function in other cancer occurrences. CENPF and FOXM1 drives the malignancy of prostate cancer^101^, CDK7 is a crucial gene in castration-resistant prostate cancer cells^70^, KIF11 is an indicator of the invasiveness of prostate cancer^73,74^, RAD51 is overexpressed in invasive prostate cancer^77^, and MYC can promotes prostate cancer development^80^. CCNE2 and BUB1B promotes the proliferation and migration of prostate cancer cells^69,79^. Additionally, ORC6 shows a positive correlation with T-regulated cell immune infiltration in prostate cancer tissues^75^, and PBK also drives prostate cancer^76^. These genes all hold potential as targets for diagnosis, prognosis, and treatment in prostate cancer. For instance, silencing TTK can inhibit the proliferation and progression of prostate cancer^78^, and KIF11 inhibitors have been developed as chemotherapy drugs for treating various cancers^73,74^. Moreover, ECT2 and GINS2 have roles in breast cancer^81,82^, RAD51AP1 plays a role in ovarian cancer^84^, RFC3 is involved in gastric and colorectal cancer^85^, and RFC4 is associated with oral tongue squamous cell carcinoma^86^, Notably, GINS2 and TUBB4B are involved in regulating cancer stem cells, PARD6A plays a role in epithelial-mesenchymal transition^87^, and RAD51AP1 promotes cancer development^84^. The roles of these genes in prostate cancer lineage plasticity should be further investigated.

CNV data was the predominant source of genes in Function Module 6 (Fig. 2H), suggesting the importance of CNV in prostate cancer lineage plasticity. Therefore, a CNV analysis of LEGs in prostate cancer was conducted, and significant CNV alterations in LEGs were identified (Fig. 5D). These provide further evidence that CNVs contribute to the driving force of prostate cancer lineage plasticity. Considering that gene expression is regulated by TFs, we identified the TFs with transcriptional activity in prostate cancer samples that can influence the expression of multiple LEGs (> 14). There are 10 such TFs, including RORC, AR, LIN28B, IRF9, NKX2-5, IRF3, RUNX1, TRPS1, FOXO1, and TP63 (Fig. 5E,F). These TFs have all been reported to play roles in prostate cancer. RORC, AR, LIN28B, IRF9, and IRF3 exert promotive roles in prostate cancer. RORC drives the expression of AR in prostate cancer^89^. AR is a very important gene in castration-resistant prostate cancer (CRPC), with 80% of CRPCs carrying elevated AR gene copy numbers and approximately 30% of CRPCs having high-level AR gene amplification^90^. LIN28B is a potential target for the treatment of neuroendocrine prostate cancer (t-NEPC)^102^, as it promotes the development of the neuroendocrine prostate cancer^91^. IRF9 and IRF3 play roles in prostate cancer progression^92,94,95^. Additionally, the transcriptional activity of RORC, AR, LIN28B, IRF9, and IRF3 is positively correlated with LEGs (Fig. 5F). Combining existing reports with the analysis presented in this study, it can be inferred that RORC, AR, LIN28B, IRF9, and IRF3 promoted prostate cancer lineage plasticity through the RTK/RAS pathway. NKX2-5, RUNX1, TRPS1, FOXO1, and TP63 play a inhibitory roles in the development of prostate cancer. NKX2-5 exhibits anti-cancer activity in the process of prostate cancer development^93^, and elevated RUNX1 expression in prostate cancer patients tends to be associated with a more favorable clinical prognosis^96^. TRPS1 may participate in oxidative stress-induced apoptosis in androgen-independent DU145 prostate cancer cells^103^. TP63 regulates the expression of p53, which can inhibit prostate cancer lineage plasticity, meaning it could potentially contribute to the inhibition of prostate cancer lineage plasticity^8^. FOXO1can also restrain the migration and invasion of prostate cancer cells^98^. The transcriptional activities of NKX2-5, RUNX1, TRPS1, FOXO1, and TP63 are negatively correlated with LEGs (Fig. 5F), suggesting these suppress prostate cancer lineage plasticity through the RTK/RAS pathway. The process of the RTK/RAS pathway driving prostate cancer lineage plasticity is a complex interplay involving multiple TFs, with TP63, FOXO1, and RORC potentially playing more crucial roles due to their relatively high activity levels. TP63 and FOXO1 inhibit prostate cancer lineage plasticity and RORC promotes it.

Therefore, based on multi-omics data of prostate cancer, we have identified Function Modules associated with prostate cancer lineage plasticity, and the RTK/RAS signaling pathway has been found to promote prostate cancer lineage plasticity. Both CNV and TFs contribute to this process.

## Conclusions

In this study, we identified 6 functional modules based on multiple omics datasets from prostate cancer obtained from the TCGA and SRA databases. Analysis of the functional modules revealed that Module 6 has a positive enrichment with both prostate cancer lineage plasticity and the RTK/RAS signaling pathway. The CNVs and TFs, such as *TP63*, *FOXO1*, and *RORC*, promote the RTK/RAS pathway-driven prostate cancer lineage plasticity by upregulating the LEGs. This research revealed the crucial role of the RTK/RAS signaling pathway in promoting prostate cancer lineage plasticity, providing a comprehensive list of potential therapeutic targets for prostate cancer lineage plasticity. Furthermore, it offers valuable insights for studying lineage plasticity in other types of malignancies.

## Methods

### Joint analysis of AR ChIP-seq data and transcriptome data in the TCGA database

Twenty raw AR ChIP-seq datasets were downloaded from NCBI's SRA database and named GSE70079^3^, including 13 from cancer patients and 7 from normal samples. Adaptor sequences were removed using Trimmomatic^104^, followed by removal of low-quality bases using FASTX-Toolkit^105^, and lastly quality-control metrics were obtained using FastQC^106^. Resulting filtered data was aligned to a reference genome using Bowtie2^107^. Then, peaks were called using MACS^108^, and annotated with HOMER^109^ to obtain the AR-regulated genes. Raw count transcriptome data were obtained from the TCGA database (https://www.cancer.gov/tcga) and analyzed for differential gene expression using edgeR^110^. Subsequently, the AR-regulated genes were merged with differentially expressed genes (DEGs) from the transcriptome to detect common genes. Finally, clusterProfiler^111^ was used to perform GO and KEGG (https://www.kegg.jp/kegg/kegg1.html)^112^ enrichment analysis on these common genes with a significance level of *p-value* < 0.05 and *q-value* < 0.05.

### Univariate and multivariate Cox analyses of LncRNA and functional predictions

LncRNA data was downloaded and extracted from the transcriptome of PCa in the TCGA database, and differential expression lncRNAs were obtained using edgeR. Fifteen lncRNAs (*p* < 0.01) were obtained based on the differential expression and clinical data downloaded from the TCGA database using the survrval package^113^ for univariate Cox analysis. 15 lncRNAs were identified through univariate Cox analysis, and subsequently, 5 lncRNAs were finalized after multivariate Cox analysis. Then, the accuracy of the model was validated through ROC curves^114^ based on TCGA and GEO^115^ test sets. After performing a correlation analysis between lncRNAs and mRNAs, two lncRNAs were ultimately obtained that exhibited correlation. These two lncRNAs were then subjected to GO and KEGG enrichment analysis using clusterProfiler^111^, with *p-value* < 0.05 and *q-value* < 0.05.

### Analysis of extraprostatic extension and intraprostatic tumors data

Based on the definitions of the American Joint Committee on Cancer (AJCC) tumor, node, metastasis (TNM) system, prostate cancer staged T3-T4 was classified as extraprostatic extension, and stage T2 was classified as intraprostatic tumors. DEGs were identified based on these two classifications. Utilizing the Gleason primary and secondary scores in the TCGA clinical information, samples were classified according to the Gleason grading system (Grade Group 1 = Gleason 6 or less; Grade Group 2 = Gleason 3 (primary) + 4 (secondary) = 7; Grade Group 3 = Gleason 4 + 3 = 7; Grade Group 4 = Gleason 8; Grade Group 5 = Gleason 9–10). Employing the five Gleason grades, genes associated with Gleason grading were extracted from DEGs, and subsequent GO and KEGG enrichment analyses were performed.

### SNV data analysis from the TCGA database

Based on clinical information of extraprostatic extension and the Gleason grade in the TCGA database, a SNV waterfall plot was generated using GenVisR package^116^. The formula for enrichment analysis (hypergeometric test) is as follows:

1 $$P(X = k) = \tfrac{{\left( \begin{subarray}{l} M \\ k \end{subarray} \right)\left( \begin{subarray}{l} N - M \\ n - k \end{subarray} \right)}}{{\left( \begin{subarray}{l} N \\ n \end{subarray} \right)}}$$

N represents the total population size, M represents the number of elements in the population with a specific attribute, n represents the number of draws, and k represents the number of hits for the element with the specific attribute. Based on Formula (1), perform enrichment analysis on TP53 mutation samples among high mutation samples. Genes related to SNVs were obtained from prostate cancer SNV and transcriptome data, and GO and KEGG enrichment analysis was performed on these.

### Analysis of CNV and Hi-C data

Based on CNV data from TCGA, gene copy numbers were obtained using the formula 2^(Segment + 1). Based on the results of gene copy numbers, values greater than 3.5 were defined as 2 (amplification), values greater than 2.5 and less than or equal to 3.5 were defined as 1 (Single gain), values less than 0.5 were defined as -2 (Double deletion), values greater than or equal to 0.5 and less than 1.5 were defined as -1 (Single deletion), and the remaining values were defined as 0 (normal). This ultimately led to the construction of a CNV matrix with four types of mutations and one normal type. Genes with significant differences in CNV between tumor and normal samples were identified through chi-square tests. Based on prostate cancer transcriptomic and CNV data, genes whose expression were significantly affected by CNV were identified with the Kruskal–Wallis test. Utilizing the list of genes with significant differences in CNV, the count of samples with the most prevalent mutation type within this list was extracted from the CNV matrix, and the proportion of these samples in relation to tumor samples was computed. GO and KEGG enrichment analyses were performed on genes with significant CNV differences to obtain results related to functions and pathways. Obtain prostate cancer Hi-C data^117^, and after quality control^118^, use Hic-Pro^119^ and Juicer^120^ to identify differential loops between prostate cancer and normal samples. Ultimately, CNV and loop circos plots were generated using the RCircos^121^ package.

### Analysis of methylation data from the TCGA database

Based on prostate cancer methylation and transcriptome data from the TCGA database, methylation-driven genes were obtained using the MethyMix package^122^, and GO and KEGG enrichment analysis were performed on these.

### Protein–protein interaction network analysis and RT-qPCR experiments

Protein–protein interaction data for prostate cancer-related genes were obtained from the STRING database. A network was visualized using the software Cytoscape (3.9.1 version; https://cytoscape.org/index.html)^123^, and the core interacting protein network was extracted.

Total RNA from all samples was extracted using the TRIzol reagent (Invitrogen, USA), and genomic DNA contamination was removed using DNase I (Promega, USA). RNA yield was measured using a NanoDrop ND-2000 (Thermo Fisher Scientific, USA), and integrity was assessed on a 1% agarose gel. cDNA was synthesized using oligo-dT primers and SuperScript III (Takara, JP). Real-time Quantitative PCR (RT-qPCR) was performed using the Plus real-time PCR system (Applied Biosystems, USA). mRNA RT-qPCR was carried out using the SYBR Prime ScriptTM RT-PCR kit (Takara, JP). Data were analyzed using the relative 2^−ΔΔCT^ method^124^. RT-qPCR primer sequences are listed in Supplementary Table S22.

### Functional and pathway clustering of multi-omics data

Based on the results of GO and KEGG enrichment analysis from multi-omics data, a matrix of kappa values^125–127^ is constructed using gene pairs. Then, based on the kappa value matrix, a clustering tree is constructed using the bottom-up Neighbor Joining Algorithm^128^. The clustering results of functions and pathways were visualized using the R package ggtree^129^. The information for each cluster is counted using the UpSetPlot package^130^, and then a gene list is constructed based on the gene set of each cluster and the logFC values from the transcriptomic data analysis results. Finally, each cluster is annotated using the GSEA algorithm^131^, and the HALLMARK and SU2C databases^9^. Furthermore, protein expression identification of the prostate cancer genes in the clustering analysis results was performed using the protein data from the Human Protein Atlas database.

Transcriptome data for CRPC^132^ and NEPC^133^ were obtained from cBioPortal^134^ (Supplementary Fig. S6), and differentially expressed genes between CRPC, NEPC, and TCGA were identified using limma^135^. Based on the differentially expressed genes, CRPC and NEPC scores for TCGA were calculated using the gsva package^136^. Then, using the gene sets of six functional modules and the gsva package, sample scores for TCGA, CRPC, and NEPC were calculated. Finally, the relationship between CRPC, NEPC, and functional module 6 was determined using fgsea^137^.

### Analysis of the relationship between prostate *cancer* lineage plasticity and RTK/RAS signaling pathways

Based on the GSEA results, clusters associated with stem cell function were selected. Stemness-related genes^66,67^, multi-lineage state-related genes^7^, 10 classic cancer pathways^16^, and the GSEA algorithm were used to analyze the relationship between stem cell function-related clusters and both prostate cancer lineage plasticity and classic cancer pathways.

Firstly, the ssGSEA algorithm^131^ was used to quantify the plasticity phenotype scores of prostate cancer samples from the TCGA database based on the prostate cancer lineage plasticity gene set^7,65,66^. Then, we employed WGCNA^138^ to compute the relationship between the multi-lineage state phenotype of these samples and the gene set of Function 6 module (stemness module). We retained the positive correlation values between the two. And finally, the relationship between RTK/RAS signaling pathways^16,139,140^ and prostate cancer lineage plasticity was analyzed employing GSEA.

### Analysis of the transcriptional expression, copy number variation, and transcription factor activity of leading-edge genes

Based on the GSEA analysis results for stemness, shTP53/RB1 + Enz, and RTK/RAS, 19 leading-edge genes (LEGs) associated with prostate cancer lineage plasticity and the RTK/RAS signaling pathway were obtained. Single-cell transcriptomic data were retrieved from the SRA database, and the single-cell expression matrix was produced using cutadapt^118^, hisat2^141^, and featureCounts^142^. Cell population analysis was subsequently conducted using Seurat^143^. Based on the analysis results of prostate cancer transcriptomic data from the TCGA database and single-cell transcriptomic data, the expression profiles of the LEGs were displayed. The expression profile of LEGs in prostate cancer cells was investigated based on the transcriptome data from TCGA prostate cancer database and single-cell transcriptome data^68^. The copy number variation of LEGs in prostate cancer is explored based on the copy number variation data in the TCGA and SU2C databases. TFs that can influence the expression of LEGs were obtained from the GPSAdb database^88^. The activity of these TFs was inferred using the reconstruction of accurate cellular network algorithm based on the ARACNe-AP adaptive partitioning strategy^144^ and the analytic rank-based enrichment analysis algorithm based on VIPER scores^145^.

### Ethics approval and consent to participate

This study was approved by the Ethics Committee of the Wuxi No.2 People's Hospital (NO: 2023Y-27).

### Supplementary Information

Supplementary Figure S1.

Supplementary Figure S2.

Supplementary Figure S3.

Supplementary Figure S4.

Supplementary Figure S5.

Supplementary Figure S6.

Supplementary Tables.

## Data Availability

The original contributions presented in the study are included in the article/supplementary data, further inquiries can be directed to the corresponding author.

## Abbreviations

CNVs: Copy number variations
AR: Androgen receptor
NEPC: Neuroendocrine Prostate Cancer
CRPC: Castration-resistant prostate cancer
MSPC: Mesenchymal and stem-like prostate cancer cells
EMT: Epithelial-mesenchymal transition
DEGs: Differentially expressed genes
LEGs: Leading-edge genes
