# Supplementary material for: Comprehensive data mining reveals RTK/RAS signaling pathway as a promoter of prostate cancer lineage plasticity through transcription factors and CNV
Source: Sci Rep. 2024 May 22;14:11688. doi: 10.1038/s41598-024-62256-z (PMC11111877; doi:10.1038/s41598-024-62256-z)
Supplement: Supplementary file 1 — Supplementary Figure S1. [file 41598_2024_62256_MOESM1_ESM.pdf]

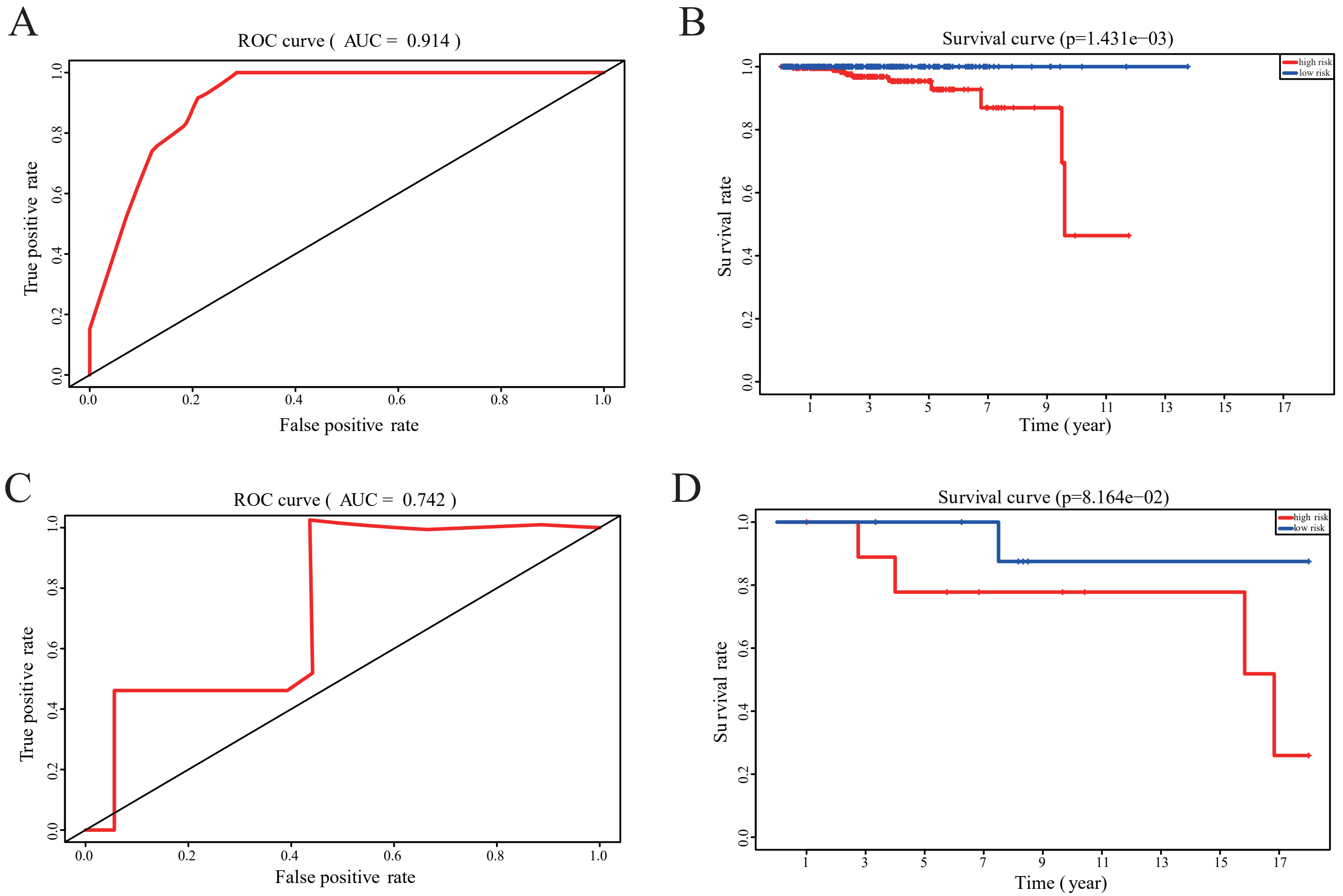

Supplementary Figure S1. ROC and survival curves of the lncRNAs prognostic model: (A) and (B) are the test results from the TCGA database; (C) and (D) are the test results from the GEO database.
