# Supplementary material for: Comprehensive data mining reveals RTK/RAS signaling pathway as a promoter of prostate cancer lineage plasticity through transcription factors and CNV
Source: Sci Rep. 2024 May 22;14:11688. doi: 10.1038/s41598-024-62256-z (PMC11111877; doi:10.1038/s41598-024-62256-z)
Supplement: Supplementary file 2 — Supplementary Figure S2. [file 41598_2024_62256_MOESM2_ESM.pdf]

	CRPC_Score = high (n=217)	CRPC_Score = low(n=213)	NEPC_Score = high(n=221)	NEPC_Score = low(n=209)	P Value
**T**					
T2a	4 (1.8%)	6 (2.8%)	3 (1.4%)	7 (3.3%)	1.4e-06*
T2b	2 (0.9%)	6 (2.8%)	3 (1.4%)	5 (2.4%)	
T2c	47 (21.7%)	84 (39.4%)	60 (27.1%)	71 (34.0%)	
T3a	70 (32.3%)	75 (35.2%)	63 (28.5%)	82 (39.2%)	
T3b	84 (38.7%)	39 (18.3%)	84 (38.0%)	39 (18.7%)	
T4	10 (4.6%)	3 (1.4%)	8 (3.6%)	5 (2.4%)	
**N**					
N0	161 (74.2%)	188 (88.3%)	166 (75.1%)	183 (87.6%)	1.7e-05*
N1	56 (25.8%)	25 (11.7%)	55 (24.9%)	26 (12.4%)	
**Gleason grade**					
Group 1	6 (2.8%)	20 (9.4%)	10 (4.5%)	16 (7.7%)	1.1e-07*
Group 2	43 (19.8%)	75 (35.2%)	49 (22.2%)	69 (33.0%)	
Group 3	46 (21.2%)	45 (21.1%)	45 (20.4%)	46 (22.0%)	
Group 4	28 (12.9%)	33 (15.5%)	29 (13.1%)	32 (15.3%)	
Group 5	94 (43.3%)	40 (18.8%)	88 (39.8%)	46 (22.0%)	
**Drug treatment**					
False	169 (77.9%)	191 (89.7%)	175 (79.2%)	185 (88.5%)	4.8e-04*
True	48 (22.1%)	22 (10.3%)	46 (20.8%)	24 (11.5%)	

* significant

Supplementary Figure S2. Clinical Information Table for TCGA Cohort Stratified by High and Low CRPC and NEPC Scores.
