# Supplementary material for: Comprehensive data mining reveals RTK/RAS signaling pathway as a promoter of prostate cancer lineage plasticity through transcription factors and CNV
Source: Sci Rep. 2024 May 22;14:11688. doi: 10.1038/s41598-024-62256-z (PMC11111877; doi:10.1038/s41598-024-62256-z)
Supplement: Supplementary file 3 — Supplementary Figure S3. [file 41598_2024_62256_MOESM3_ESM.pdf]

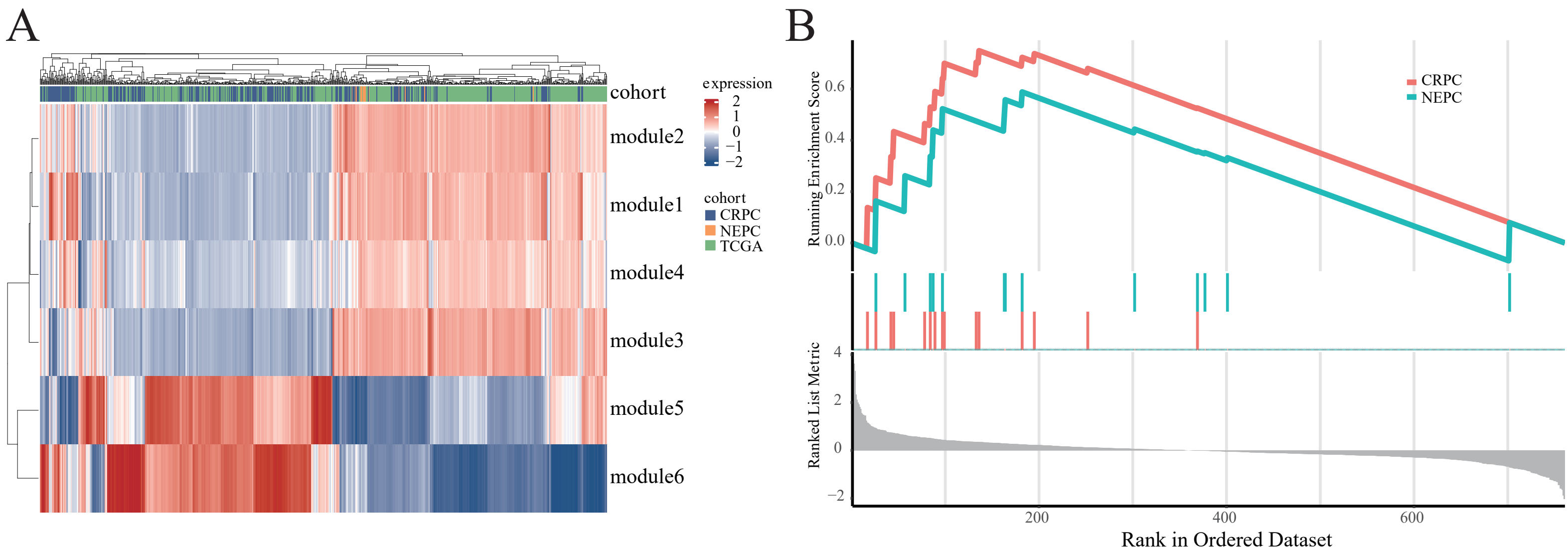

Supplementary Figure S3. Relationship Chart of Functional Modules with TCGA, CRPC, and NEPC Cohorts. (A) Heatmap of the scoring of TCGA, CRPC, and NEPC cohorts by six functional modules. (B) Functional module 6 shows a positive correlation with CRPC and NEPC characteristics.
