# Supplementary material for: Comprehensive data mining reveals RTK/RAS signaling pathway as a promoter of prostate cancer lineage plasticity through transcription factors and CNV
Source: Sci Rep. 2024 May 22;14:11688. doi: 10.1038/s41598-024-62256-z (PMC11111877; doi:10.1038/s41598-024-62256-z)
Supplement: Supplementary file 4 — Supplementary Figure S4. [file 41598_2024_62256_MOESM4_ESM.pdf]

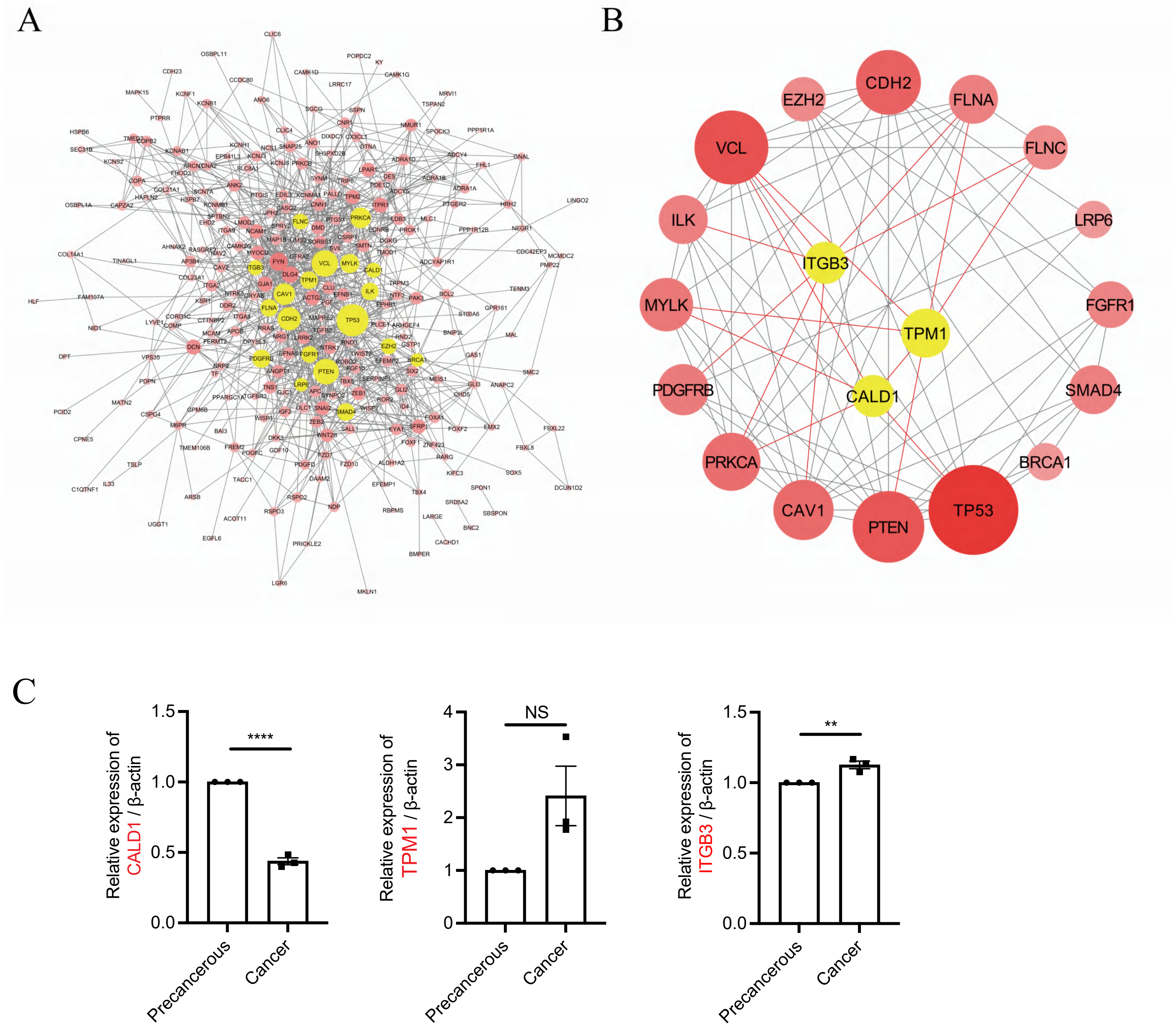

Supplementary Figure S4. The protein-protein interaction (PPI) network of intersection genes from the six datasets in prostate cancer. (A) Global PPI network; (B) Core PPI network; (C) RT-qPCR of TPM1, ITGB3, and CALD1.
