# Supplementary material for: Comprehensive data mining reveals RTK/RAS signaling pathway as a promoter of prostate cancer lineage plasticity through transcription factors and CNV
Source: Sci Rep. 2024 May 22;14:11688. doi: 10.1038/s41598-024-62256-z (PMC11111877; doi:10.1038/s41598-024-62256-z)
Supplement: Supplementary file 5 — Supplementary Figure S5. [file 41598_2024_62256_MOESM5_ESM.pdf]

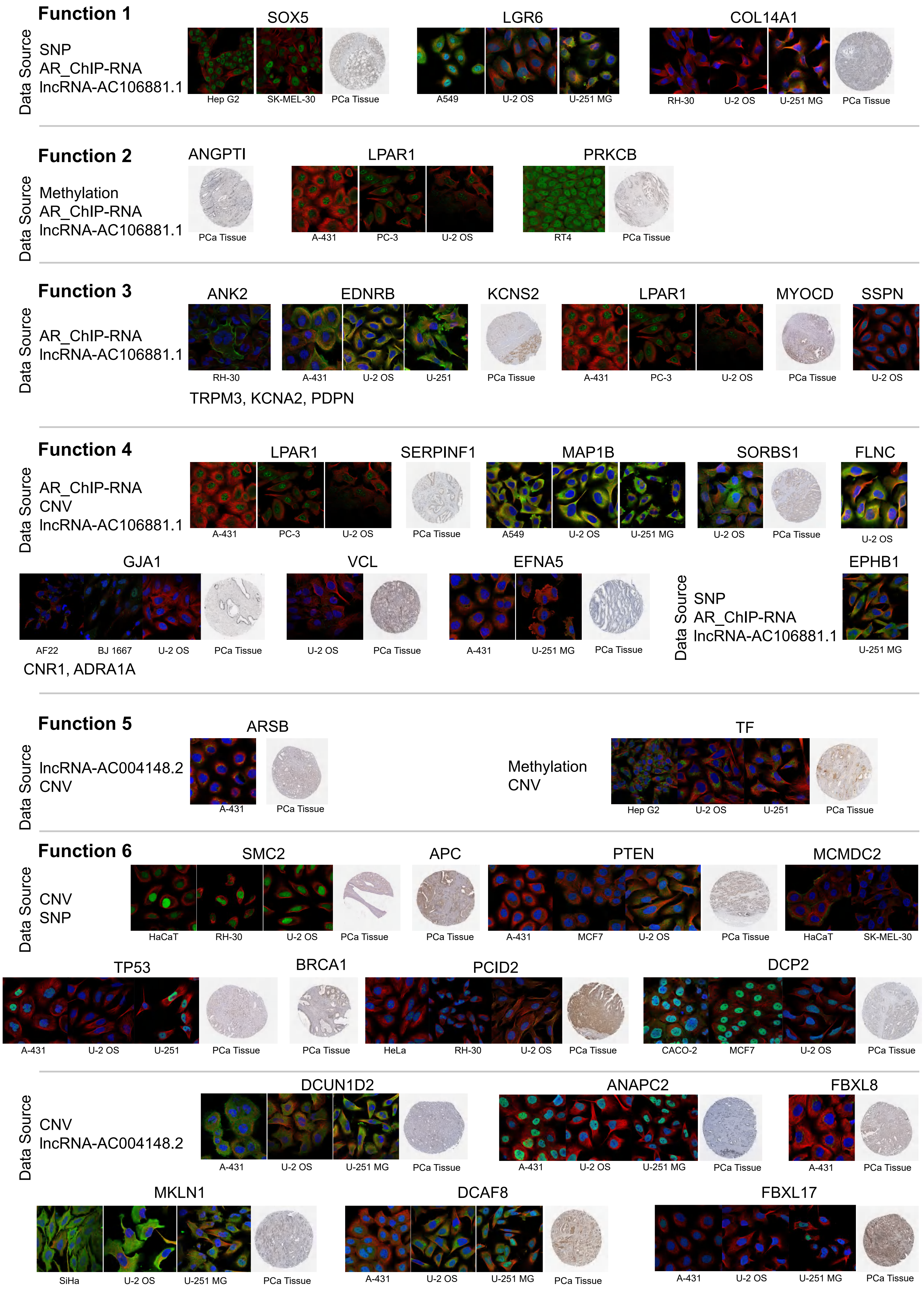

Supplementary Figure S5. Cellular proteomics (Human Protein Atlas) in prostate cancer tissue and cancer cell lines involving 42 intersecting genes derived from multiple datasets in the six Functional Modules.
