# Supplementary material for: Comprehensive data mining reveals RTK/RAS signaling pathway as a promoter of prostate cancer lineage plasticity through transcription factors and CNV
Source: Sci Rep. 2024 May 22;14:11688. doi: 10.1038/s41598-024-62256-z (PMC11111877; doi:10.1038/s41598-024-62256-z)
Supplement: Supplementary file 6 — Supplementary Figure S6. [file 41598_2024_62256_MOESM6_ESM.pdf]

Project	Identifier	Data
TCGA_PRAD	TCGA	https://portal.gdc.cancer.gov/
SU2C_2019	CRPC	https://github.com/cBioPortal/datahub/tree/master/public/prad_su2c_2019
WCM_2016	CRPC and NEPC	https://github.com/cBioPortal/datahub/tree/master/public/nepc_wcm_2016

Supplementary Figure S6. Table of Data Source Information for the TCGA, CRPC, and NEPC Cohorts
